# Sleep inertia, daytime sleepiness, and falls in older adults of the Wisconsin Sleep Cohort

**DOI:** 10.1093/sleepadvances/zpag106

**Published:** 2026-09-29

**Authors:** Jesse D Cook, Jodi H Barnet, Jessica J Love, Laurel A Ravelo, Annaliise J Hayrynen, Amanda Rasmuson, Erika W Hagen, Paul E Peppard, David T Plante

**Affiliations:** University of Wisconsin-Madison School of Medicine and Public Health, Department of Psychiatry, Madison, WI, United States; University of Wisconsin-Madison School of Medicine and Public Health, Department of Population Health Sciences, Madison, WI, United States; University of Wisconsin-Madison School of Medicine and Public Health, Department of Psychiatry, Madison, WI, United States; University of Wisconsin-Madison, Department of Psychology, Madison, WI, United States; University of Wisconsin-Madison School of Medicine and Public Health, Department of Population Health Sciences, Madison, WI, United States; Meadville Medical Center, Neurology, Meadville, PA, United States; University of Wisconsin-Madison School of Medicine and Public Health, Department of Population Health Sciences, Madison, WI, United States; University of Wisconsin-Madison School of Medicine and Public Health, Department of Population Health Sciences, Madison, WI, United States; University of Wisconsin-Madison School of Medicine and Public Health, Department of Population Health Sciences, Madison, WI, United States; University of Wisconsin-Madison School of Medicine and Public Health, Department of Psychiatry, Madison, WI, United States; University of Wisconsin-Madison, Department of Psychology, Madison, WI, United States

**Keywords:** falls, sleep inertia, daytime sleepiness, aging, sleep health, older adults

## Abstract

**Study Objectives:**

Examine associations between sleep inertia, daytime sleepiness, and falls in older adults of the Wisconsin Sleep Cohort (WSC).

**Materials and Methods:**

Cross-sectional data from WSC participants were used. Participants completed the Sleep Inertia Questionnaire (SIQ) and Epworth Sleepiness Scale (ESS). Elevated sleep inertia (ESI) was defined as the upper quartile (SIQ > 37), while ESS > 10 defined excessive daytime sleepiness (EDS). Falls outcomes included self-reported recurrent (<2 vs 2+) and injurious (no vs yes) falls over the previous year. Logistic regressions examined associations with falls outcomes. Fully adjusted analyses included demographics and outcome-specific sleep, psychiatric, medical, physical, and cognitive covariates. Secondary analyses explored associations with SIQ subscales.

**Results:**

The recurrent falls sample included 392 older adults (74.5 ± 6.6 years; 44.9% female), while 162 participants with ≥1 fall comprised the injurious falls sample (75.4 ± 6.6 years; 45.1% female). In unadjusted analyses, higher SIQ scores and ESI were significantly associated with recurrent and injurious falls; however, in fully adjusted analyses, the associations remained significant only for recurrent falls (SIQ: OR = 1.04, 95% CI = 1.00–1.07; ESI: OR = 2.00, 95% CI = 1.07–3.73). Higher ESS scores and EDS were significantly associated with recurrent falls in unadjusted analyses, but not in fully adjusted analyses. Neither ESS nor EDS was associated with injurious falls. In SIQ subscale analyses, Physiological symptoms were most strongly associated with recurrent falls, whereas Cognitive symptoms were most strongly associated with injurious falls.

**Conclusions:**

Sleep inertia may represent an important, under-recognized risk factor for falls in older adults.

## Teaser

Daytime sleepiness and sleep inertia are both safety hazards that may contribute to fall risk in older adults; however, their relationships with fall outcomes are understudied. In this investigation, greater sleep inertia severity was consistently associated with increased odds of recurrent falls, and there was some evidence for a similar relationship with injurious falls. The results from this investigation also provided some further support for daytime sleepiness as a risk factor for recurrent falls, yet daytime sleepiness was not associated with injurious falls. These findings demonstrate that sleep inertia may be an underappreciated but important sleep health characteristic in the context of fall risk, guiding future research that explores its role in fall prevention and risk management.

## Graphical abstract

**Graphical abstract:**
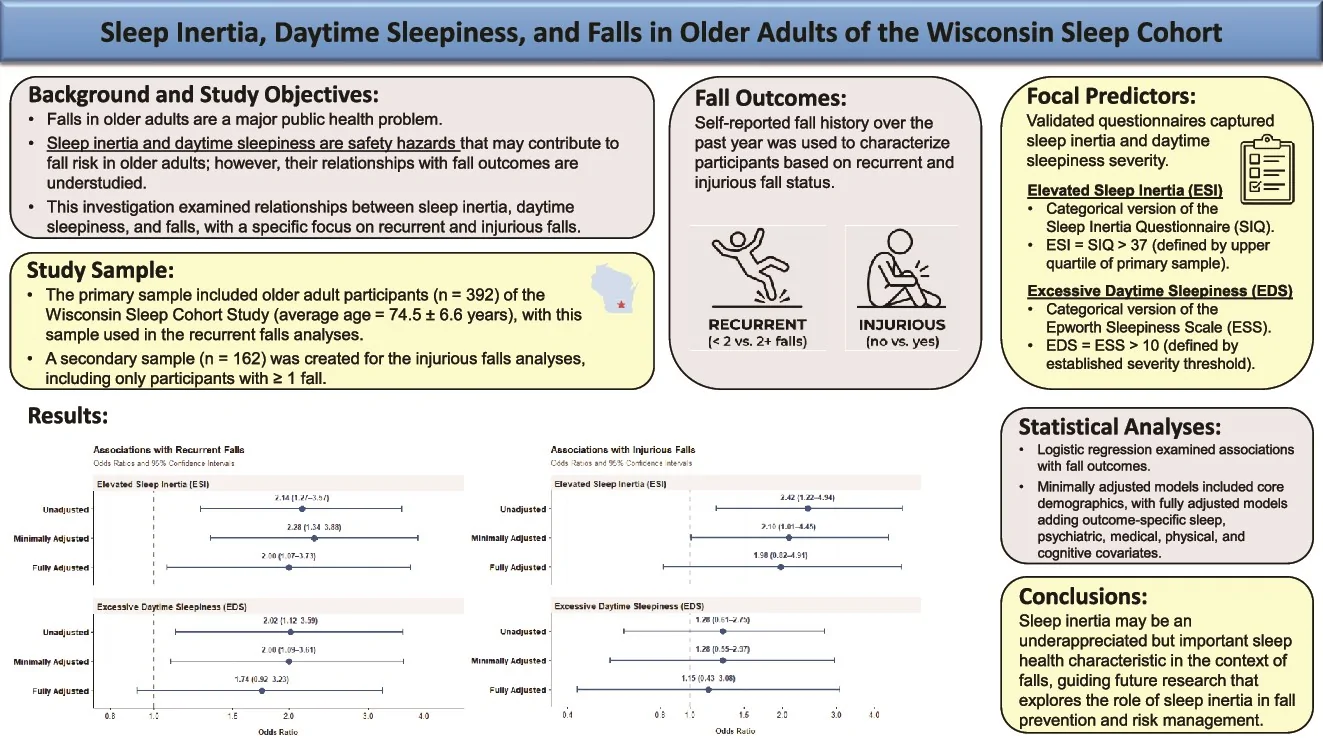

## Introduction

Falls are highly prevalent among older adults, with more than one in four experiencing a fall annually [1, 2]. Falls can result in serious injuries, including bone fractures and head trauma [3] and are the leading cause of unintentional injury-related death in older adults [4]. The societal burden of falls is substantial, with annual medical costs associated with fatal and nonfatal falls in the United States estimated at $50 billion [5]. Identifying modifiable risk factors for falls in older adults is critical for improving risk assessment, prevention, and management strategies.

Sleep health is increasingly recognized as a modifiable risk factor for a range of health outcomes [6, 7], including falls [8]. Multiple sleep-related characteristics have been associated with falls, including short and long sleep duration [9], sleep apnea [10], insomnia symptoms [11], daytime napping [12], and poor sleep quality [13]. Excessive daytime sleepiness (EDS), a consequence of sleep deficiencies, disruptions, and disorders [14], is a well-established safety hazard [15] that has also been linked to falls [13, 16–18]. However, prior work has largely focused on the association between EDS and the number of falls, leaving uncertainty regarding the relationship between EDS and injurious falls.

Sleep inertia, a transient period of cognitive, physiological, and/or psychological impairment occurring upon awakening [19, 20], is also recognized as a safety hazard [21]. Sleep inertia is a universal experience, with many individuals experiencing manageable, mild symptoms that typically dissipate within 30 min [19]. However, symptom severity and duration vary between individuals [22], and some people experience more frequent, prolonged, and/or intense symptoms [20, 23]. Emerging evidence suggests that older adults may be particularly vulnerable to more severe sleep inertia [24]. In addition, the early morning hours have been identified as a high-risk time of day for more severe falls [25]. In this context, symptoms of sleep inertia, including grogginess, disorientation, slowed processing speed, impaired vigilance and motor coordination, physical lethargy, dizziness, and heightened emotional anxiety [20, 26, 27], may plausibly contribute to fall risk in older adults. Despite this potential relevance, sleep inertia remains understudied in the context of falls in older adults.

This investigation was designed to address these knowledge gaps by examining the relationships between sleep inertia, daytime sleepiness, and falls outcomes in older adults of the Wisconsin Sleep Cohort (WSC). We hypothesized that both greater sleep inertia and daytime sleepiness severity would be associated with higher odds of recurrent and injurious falls over the prior year, observable in analyses that modeled sleep inertia and daytime sleepiness both continuously and categorically. Secondary analyses further explored associations between specific dimensions of sleep inertia and fall-related outcomes. Collectively, the results of this study aim to advance the understanding of sleep-related risk factors for falls in older adults and to inform future efforts to improve fall prevention and management.

## Materials and methods

### Dataset description, analytic samples, and ethical approval

Cross-sectional data from the WSC Study were utilized for this investigation. Detailed descriptions of the study design, methodology, and objectives of the WSC have been published elsewhere [28, 29]. Briefly, the WSC is a landmark, longitudinal epidemiologic study of sleep health in community-dwelling adults of Wisconsin, United States. Data collection began in the late 1980s, with a cohort of participants established from a two-stage sampling scheme. First, full-time state employees (ages 30–60) from four state agencies in south-central Wisconsin were mailed a short survey about their sleep, general health, and demographics. At that time, the primary focus of the WSC centered on investigating the natural history of sleep disordered breathing, which guided the recruitment of a stratified sample from the responders that over-sampled participants at high-risk for sleep apnea. Since its genesis, participants have been followed longitudinally, completing study visits approximately every 4 years. The scope and aims of the WSC have evolved over time, expanding to other sleep disorders and sleep health more broadly. Currently, the cohort is composed predominantly of older adults and data collection is ongoing, serving as a valuable resource for aging-related research.

Measures included in the WSC have evolved over time, with the Sleep Inertia Questionnaire (SIQ) [27] added to the study assessments in 2019. For the present analyses, data collected between 2019 and 2023 from non-shift-working older adults (≥60 years) at the time of assessment were used. The measures used across this investigation were collected at the same study visit for each participant. All participants in the initial sample completed both the SIQ and the Epworth Sleepiness Scale (ESS) [30] and reported information on falls over the previous year (including number of falls and fall-related injury). Based on these criteria, the initial sample consisted of 403 older adults, with these data used for analyses related to number of falls (Falls-Number). This dataset was further subset to create an injurious-specific dataset (Falls-Injury), including only participants who reported at least one fall during the prior year (*n* = 165). Both samples were slightly reduced after determining the covariates for the fully adjusted model (see section Additional Covariates section), retaining only participants with complete cases across all covariates. This resulted in analytic samples of 392 for Falls-Number and 162 for Falls-Injury.

All study procedures were approved by the University of Wisconsin-Madison Health Sciences Institutional Review Board and written informed consent was obtained from all individuals as part of their participation in the WSC.

### Collected measures and variable preparation

#### Predictor variables: SIQ, SIQ subscales, and ESS

The SIQ and ESS were prepared continuously and categorically for statistical analyses.

The SIQ is a validated, 23-item self-report measure of sleep inertia [27]. The first 21 items are scored on a 5-point Likert scale (1–5) and summed to yield the total SIQ score, with higher scores indicating greater sleep inertia severity. Developed as a trait-level measure of sleep inertia, the SIQ’s instructions guide responders to complete each item in the context of a typical morning in the past week. At the time of this investigation, no established threshold existed for defining excessive sleep inertia in a community-based sample. Given this, elevated sleep inertia (ESI) was categorized using an upper-quartile cutoff in the primary analytic sample (Q4; SIQ > 37), with participants from the lower three quartiles (Q1–Q3) combined into a lower symptom severity reference group. This categorization approach afforded the ability to examine relationships in a higher symptom burden group, while maximizing the sample size of the reference group. As a complement to the categorical SIQ (Q4 vs Q1–Q3), the total SIQ score was evaluated continuously to assess whether incremental increases in sleep inertia severity were linearly associated with fall outcomes. The 21 scored SIQ items comprise four established subscales [27], reflecting different components of sleep inertia: Cognitive (five items), Physiological (eight items), Emotional (three items), and Responses to Sleep Inertia (five items; “Responses”). For secondary, exploratory analyses, subscale total scores were computed as continuous predictors, along with dichotomized, categorical versions using an upper-quartile approach that mirrored the full SIQ: Cognitive > 9, Physiological > 15, Emotional > 4, and Responses > 9.

Daytime sleepiness was measured using the ESS, a widely used and validated self-report questionnaire that is considered the gold standard for assessing subjective daytime sleepiness [30]. Designed to assess habitual daytime sleepiness, the scale instructions guide responders to rate their likelihood of dozing off or falling asleep in routine situations, without providing a specific timeframe to anchor these responses. The ESS consists of eight items, with each item scored on a 4-point Likert scale (0–3), yielding scores ranging from 0 to 24. EDS was defined using the established cutoff of ESS > 10 [30, 31]. The total ESS score was also used as a continuous predictor to evaluate the linear relationship between daytime sleepiness symptom severity and fall outcomes.

#### Outcome variables: Falls-Number and Falls-Injury

All participants completed two items about falls over the previous year. Participants self-reported the number of falls experienced in the past 12 months (Falls-Number), with the response options of “0,” “1,” “2,” “3–4,” “5–7,” or “8+.” For use as an outcome variable, Falls-Number was dichotomized to distinguish participants with fewer than two falls from those with recurrent falls (<2 vs ≥2 falls). A second item assessed whether participants experienced an injurious fall during the past 12 months (Falls-Injury), with binary response options of “no” or “yes.” For this item, guidance was provided directly in the survey prompt to assist in defining a fall-related injury: “For example, a bruise, cut or wound, sprain, dislocation, fracture, broken bones, back pain, head or neck injury.”

#### Additional covariates

A comprehensive collection of 21 covariates was selected from available WSC data based on their established or theoretical relevance to fall risk. These variables were categorized into core demographics and candidate covariates spanning sleep, medical, psychiatric, substance use, physical, and cognitive domains. Detailed descriptions of all 21 covariates are provided in Table 1.

**Table 1:** Description of covariates—core and candidate

Covariate	Format	Description
**Core demographics**		
Age	Continuous	Age at time of collection.
Sex	Categorical	Two-level variable reflecting self-reported sex assigned at birth (male vs female).
Body mass index (BMI)	Continuous	BMI was calculated from measured height and weight.
Education history	Categorical	Two-level variable reflecting educational attainment (high school or less vs more than high school).
**Sleep**		
Habitual sleep duration	Continuous	Self-reported weekday (WD) and weekend (WE) sleep durations were used to calculate a weighted average: ((WD^*^5) + (WE^*^2))/7.
Sleep apnea history	Categorical	Two-level variable reflecting self-reported history of sleep apnea (no vs yes).
Circadian preference	Continuous	Score from a modified, 3-item version of the Reduced Morningness-Eveningness Questionnaire (rMEQ) [37], using the last three items of the rMEQ [38].
Daytime napping frequency	Continuous	Self-reported number of days per week with at least one daytime nap.
**Medical**		
Sedative medication use	Categorical	Two-level variable reflecting self-reported current sedative use (no vs yes).
Arthritis history	Categorical	Two-level variable reflecting self-reported history of arthritis (no vs yes).
Hypertension history	Categorical	Two-level variable reflecting self-reported history of hypertension (no vs yes).
**Psychiatric**		
Depressive symptomatology	Continuous	Derived from the Zung Self-Rating Depression Scale [41] with two modifications: (1) Omission of the item assessing sexual attraction/interest (#6), and (2) exclusion of the sleep-related item (#4).
Trait anxiety symptomatology	Continuous	Derived from the State–Trait Anxiety Inventory [43], based on responses to 20 trait anxiety items.
**Substance use**		
Weekly alcohol intake	Continuous	Self-reported weekly alcohol consumption (drinks)
Daily caffeine intake	Continuous	Self-reported daily caffeine consumption (drinks)
Smoking history	Categorical	Two-level variable reflecting self-reported smoking history (never vs former/current).
**Physical**		
Dominant hand grip strength	Continuous	Mean grip strength (pounds) of the dominant hand across three trials.
Standing balance	Categorical	Derived from a modified version of the 4-stage balance test (no single foot trial). A two-level variable was formed, with participants categorized as “low risk” (held tandem for ≥10 seconds) or “at risk.” [47]
Regular exercise	Categorical	Two-level variable reflecting self-reported current engagement in regular exercise (yes vs no).
**Cognitive**		
Global cognitive status	Continuous	Total score obtained from the Telephone Interview for Cognitive Status assessment.
Memory concerns history	Categorical	Two-level variable reflecting self-reported history of memory concerns (no vs yes).

Description of 21 covariates collected for the investigation, including core demographics and candidate covariates organized by domain (sleep, medical, psychiatric, substance use, physical, and cognitive). Variable formats (continuous vs categorical) reflect how the covariates were modeled in statistical analyses and are reported across the manuscript.

The four core demographic covariates are included across all minimally and fully adjusted models: age [32] (continuous, years), sex [33] (categorical; female vs male), body mass index (BMI) [34] (continuous; kg/m [2]), and education history [32] (categorical; high school or less vs more than high school). The remaining 17 candidate covariates comprise lifestyle and health factors associated with fall risk:

- Sleep: habitual sleep duration [35] (continuous; self-report weighted nightly average [hours]), sleep apnea history [10] (categorical; yes vs no), circadian preference [36] (continuous; modified 3-item Reduced Morningness-Eveningness Questionnaire [37, 38]), and daytime napping frequency [12] (continuous; self-reported number of days with ≥1 nap per week);
- Medical: sedative medication use [39] (categorical; yes vs no), arthritis history [40] (categorical; yes vs no), and hypertension history [32] (categorical; yes vs no);
- Psychiatric: depressive symptomatology [32] (continuous; derived from a modified Zung Self-Rating Depression Scale score [41]) and trait anxiety symptomatology [42] (continuous; derived from the State–Trait Anxiety Inventory [43]);
- Substance Use: weekly alcohol intake [32] (continuous; number of drinks per week), daily caffeine intake [44] (continuous; number of drinks per day), and smoking history [32] (categorical; former/current vs never);
- Physical: dominant hand grip strength [45] (continuous; average [pounds] across three trials), standing balance [46] (categorical; modified 4-stage balance test, at risk [<10 s] vs low risk [tandem stance ≥10 s] [47]), and regular exercise [48] (categorical; no vs yes);
- Cognitive: global cognitive status [49] (continuous; Telephone Interview for Cognitive Status score [50]) and memory concerns history [51] (categorical; yes vs no).

To refine the fully adjusted models, associations between candidate covariates and falls outcomes (Falls-Number and Falls-Injury) were evaluated, with a conservative significance threshold (*p* ≤ .25) [52] used to identify covariates for retention in the fully adjusted models. Univariate logistic regressions were conducted, with continuous covariates sample mean-centered prior to modeling, except for dominant hand grip strength which was centered within sex. The associations between candidate covariates and falls outcomes are presented in Table S1.

For Falls-Number models, seven candidate covariates were retained for fully adjusted analyses, complementing the core demographics in the fully adjusted model: habitual sleep duration, sleep apnea history, circadian preference, depressive symptomatology, trait anxiety symptomatology, standing balance, and memory concerns history. For Falls-Injury, six candidate covariates were retained for the fully adjusted analyses: habitual sleep duration, sleep apnea history, circadian preference, trait anxiety symptomatology, arthritis history, and sedative medication use.

### Statistical analyses

Descriptive statistics were computed separately for the analytic samples used in the Falls-Number and Falls-Injury analyses. Continuous variables are reported as mean ± standard deviation or median [interquartile range (IQR)] for normally or nonnormally distributed data, respectively, with categorical variables reported as counts (*n*) and proportions (%). Subgroup comparisons were conducted across available characteristics in the Falls-Number (<2 vs ≥2 falls) and Falls-Injury (no injurious fall vs ≥1 injurious fall) samples using independent samples *t*-tests for normally distributed continuous variables, Mann–Whitney *U* tests for non-normally distributed continuous variables, and Pearson’s Chi-square (*Χ*^2^) tests for categorical variables. In addition, descriptive statistics were calculated across SIQ quartiles for the primary analytic dataset used in the Falls-Number analyses (Table S2). Global comparisons across the SIQ quartiles were conducted via one-way analysis of variance (ANOVA) for normally distributed continuous variables, Kruskal-Wallis tests for non-normally distributed continuous variables, or Pearson’s Chi-square (*Χ*^2^) tests for categorical variables. Additionally, age- and sex-stratified boxplots were constructed to visually depict the score distributions for total SIQ and ESS scores (Figure 1) as well as the four SIQ subscales (Figure S1).

**Figure 1:**
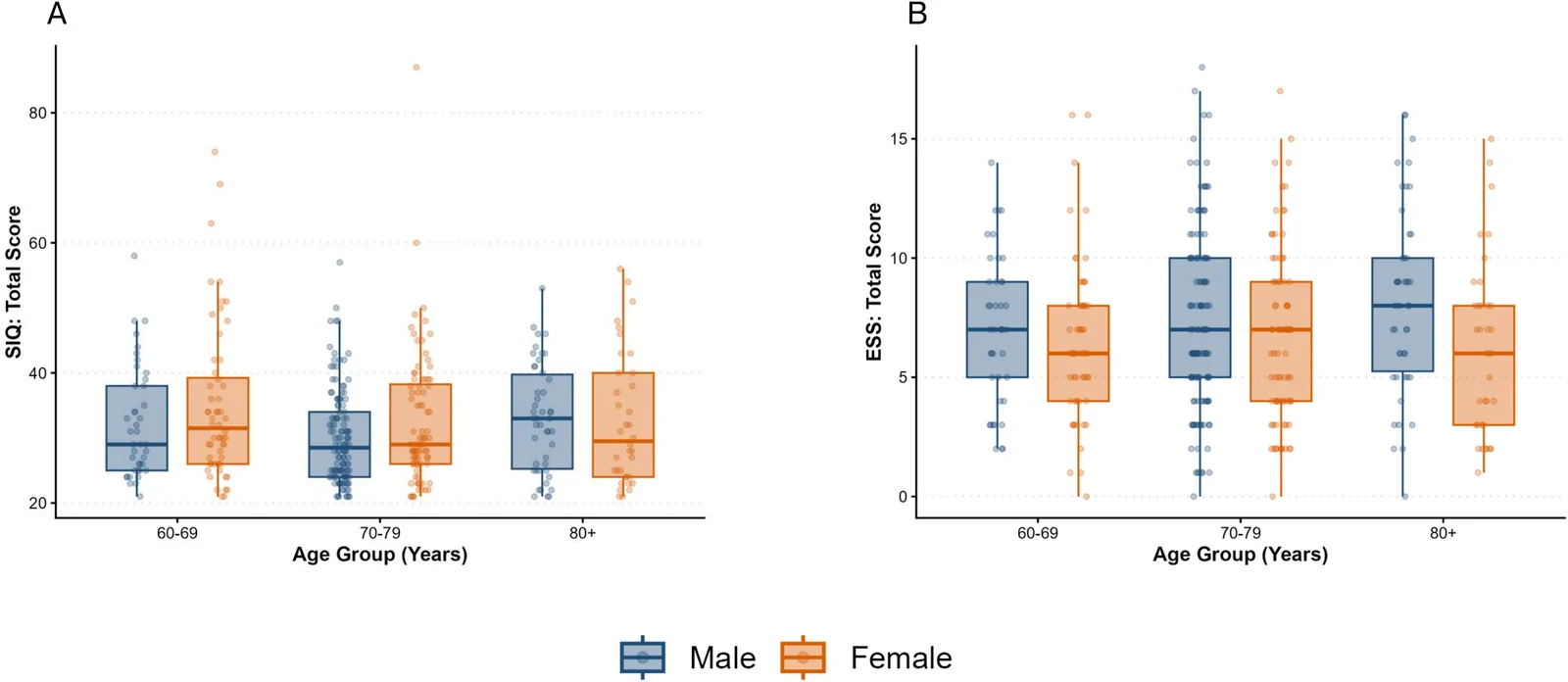
Age- and sex-stratified sleep inertia questionnaire and Epworth sleepiness scale scores. Boxplots displaying total Sleep Inertia Questionnaire (SIQ; panel A) and Epworth Sleepiness Scale (ESS; panel B) scores, stratified by age group (60–69, 70–79, and 80+) and participant sex (male or female). The horizontal line within each box indicates the median, while box bounds represent the interquartile range (IQR; 25th and 75th percentiles) and the whiskers extend no further than 1.5× IQR. Individual jittered points represent participant scores.

A series of logistic regression models were used to examine associations between predictors and falls outcomes (Falls-Number and Falls-Injury). Primary analyses examined the associations between SIQ, ESS, and falls, with SIQ and ESS modeled both continuously and categorically. Continuous formats were sample mean-centered prior to modeling. Unadjusted analyses were performed, along with minimally and fully adjusted models. For each regression, odds ratios (OR), 95% confidence intervals (CI), and corresponding *p* values are reported for the focal predictor, with confidence intervals derived using profile likelihood estimation and *p* values computed via likelihood ratio tests. Secondary analyses explored the associations between SIQ subscales and falls outcomes, using the same methodological approach employed in the primary analyses. Multicollinearity in the fully adjusted models was evaluated using the variance inflation factor (VIF), with all predictors falling below accepted thresholds (VIF < 5) [53].

Sensitivity analyses were performed exploring quartile-specific associations between sleep inertia severity and Falls-Number, using Q1 as reference group (Table S3). Global main effects across the four quartiles were evaluated, followed by pairwise comparisons assessing differences between individual quartiles (Q2–Q4) and the reference group (Q1). *P*-values for global main effects are reported alongside the OR, 95% CI, and corresponding *p* value from each pairwise comparison. A comparable sensitivity analysis was performed in the Falls-Injury sample, carrying forward the Falls-Number quartile thresholds to define the four sleep inertia severity groups, which maintains consistency with the primary methodology. Although these severity groups do not represent exact sample quartiles within the Falls-Injury sample, the labels of Q1–Q4 were maintained for these sensitivity analyses as the resulting group distribution closely resembles a quartile split. However, the smaller group sample sizes and sparse cell counts across outcomes and categorical covariates consequentially contributed to model overfitting and decreased model precision, as evidenced by noticeably wide confidence intervals. As such, the Falls-Injury sensitivity analyses are included as supplementary material for completeness (Table S4), but are not further discussed in the text and should be interpreted with caution.

Post hoc analyses were performed to explore the interrelations between sleep inertia and daytime sleepiness and to evaluate their independent associations with fall outcomes. Pearson correlation coefficients were computed between continuous SIQ and ESS scores. Additionally, logistic regression models were conducted, co-modeling both categorical sleep inertia (ESI vs no ESI) and categorical daytime sleepiness (EDS vs no EDS). Mirroring the primary analytical approach, these mutually adjusted models were evaluated without the addition of other covariates, along with minimally adjusted and fully adjusted models.

Further post hoc analyses were conducted to evaluate potential moderation by participant age (continuous) and sex (categorical). For each predictor–outcome combination, an interaction term between the predictor and the moderator (age or sex) was added to the fully adjusted model. ESS, SIQ, and SIQ subscales were modeled continuously for these post hoc analyses. The OR, 95% CI, and corresponding *p* values are reported for the interaction term from each model.

All data processing and analyses were performed in R Studio (version 4.5.2). The *car* package (version 3.1.3) was used for the VIF computation. Statistical significance was defined as *p* < .05, with *p* < .10 considered indicative of a trend-level association.

## Results

### Sample characteristics

#### Falls-Number sample characteristics

Characteristics for the Falls-Number analytic sample are presented in Table 2, including characterization of the overall sample and a subgroup comparison based on stratification by recurrent fall status (<2 vs ≥2 falls). Overall, the Falls-Number sample included 392 older adults (mean age: 74.5 ± 6.6 years) and was predominantly White (96.2%), with a slight majority of males (55.1%). The mean BMI was 31.1 ± 7.1 kg/m^2^, and most participants (80.6%) reported education beyond high school. Approximately one-quarter of participants reported recurrent falls (22.4%) over the prior year, with about one-fifth reporting at least one injurious fall (20.2%).

**Table 2:** Characteristics of the falls—number analytic sample

Characteristic	Full sample	<2 Falls	≥2 Falls	*P*
**Sample size**	392	304	88	-----
**Demographics**				
^C^Age (years), mean ± SD	74.5 ± 6.6	74.5 ± 6.5	74.6 ± 6.8	.93
^C^Sex (female), *n* (%)	176 (44.9)	142 (46.7)	34 (38.6)	.22
^C^Body mass index (kg/m^2^), mean ± SD	31.1 ± 7.1	30.7 ± 6.7	32.5 ± 8.4	.03
^C^Education history (high school or less), *n* (%)	76 (19.4)	60 (19.7)	16 (18.2)	.86
Race/ethnicity (white), *n* (%)	377 (96.2)	294 (96.7)	83 (94.3)	.48
**Falls (prior year)**				
Recurrent falls (≥2), *n* (%)	88 (22.4)	-----	-----	-----
Injurious fall (yes), *n* (%)	79 (20.2)	33 (10.9)	46 (52.3)	<.001
**Daytime sleepiness and sleep inertia**				
Epworth Sleepiness Scale				
Total score, mean ± SD	7.0 ± 3.6	6.8 ± 3.6	7.9 ± 3.8	.01
Excessive (>10), *n* (%)	65 (16.6)	43 (14.1)	22 (25.0)	.03
Sleep Inertia Questionnaire (SIQ)				
Total score, median [IQR]	30.0 [25.0, 37.0]	29.0 [25.0, 36.0]	33.0 [26.8, 41.0]	.002
Elevated (>37), *n* (%)	96 (24.5)	64 (21.1)	32 (36.4)	.005
SIQ Subscale—Cognitive				
Total score, median [IQR]	7.0 [5.0, 9.0]	6.0 [5.0, 9.0]	8.0 [5.8, 10.0]	.003
Elevated (>9), *n* (%)	91 (23.2)	62 (20.4)	29 (33.0)	.02
SIQ Subscale—Physiological				
Total score, median [IQR]	11.0 [9.0, 15.0]	11.0 [9.0, 14.0]	13.0 [10.0, 18.0]	.001
Elevated (>15), *n* (%)	92 (23.5)	59 (19.4)	33 (37.5)	.001
SIQ Subscale—Emotional				
Total score, median [IQR]	3.0 [3.0, 4.0]	3.0 [3.0, 4.0]	3.0 [3.0, 4.0]	.55
Elevated (>4), *n* (%)	60 (15.3)	45 (14.8)	15 (17.0)	.73
SIQ Subscale—Response to sleep inertia				
Total score, median [IQR]	7.0 [6.0, 9.0]	7.0 [6.0, 9.0]	8.0 [6.0, 10.0]	.08
Elevated (>9), *n* (%)	91 (23.2)	68 (22.4)	23 (26.1)	.55
**Sleep**				
^N^Habitual sleep duration (h), mean ± SD	7.5 ± 1.1	7.4 ± 1.1	7.7 ± 1.2	.008
^N^Sleep apnea history (yes), *n* (%)	130 (33.2)	95 (31.2)	35 (39.8)	.17
^N^Circadian preference (modified 3-item rMEQ score), mean ± SD	10.7 ± 2.8	10.8 ± 2.8	10.5 ± 2.7	.27
Daytime napping frequency (days per week), median [IQR]	2.0 [0.0, 4.0]	2.0 [0.0, 4.0]	2.0 [0.0, 4.0]	.47
**Medical**				
Sedative medication use (yes), *n* (%)	70 (17.9)	53 (17.4)	17 (19.3)	.80
Arthritis history (yes), *n* (%)	214 (54.7)	168 (55.4)	46 (52.3)	.69
Hypertension history (yes), *n* (%)	208 (53.5)	164 (54.3)	44 (50.6)	.62
**Psychiatric**				
^N^Depressive symptomatology (modified Zung score), mean ± SD	33.0 ± 7.5	32.6 ± 7.5	34.3 ± 7.5	.06
^N^Trait anxiety symptomatology, median [IQR]	29.0 [24.0, 36.2]	29.0 [24.0, 36.0]	31.0 [25.0, 38.0]	.12
**Substance use**				
Alcohol intake (drinks per week), median [IQR]	2.0 [0.0, 5.0]	2.0 [0.0, 5.0]	1.0 [0.0, 6.0]	.66
Caffeine intake (drinks per day), median [IQR]	2.0 [1.0, 3.0]	2.0 [1.0, 3.0]	2.0 [1.0, 3.0]	.96
Smoking history (former/current), *n* (%)	176 (44.9)	136 (44.7)	40 (45.5)	1.00
**Physical**				
Dominant hand grip strength (lbs)				
Female, mean ± SD	47.5 ± 12.3	47.1 ± 12.4	49.4 ± 12.0	.33
Male, mean ± SD	77.4 ± 20.0	78.4 ± 19.8	74.5 ± 20.4	.22
^N^Standing balance (at-risk), *n* (%)	108 (27.6)	79 (26.0)	29 (33.0)	.25
Regular exercise (no), *n* (%)	218 (55.8)	168 (55.4)	50 (56.8)	.92
**Cognitive**				
Telephone interview for cognitive status score, mean ± SD	34.9 ± 2.9	35.0 ± 2.8	34.9 ± 3.2	.80
^N^Memory concerns history (yes), *n* (%)	98 (25.0)	68 (22.4)	30 (34.1)	.04

rMEQ, Reduced Morningness-Eveningness Questionnaire; Zung, Zung Self-Rating Depression Scale.

Characteristics for the sample used in the number of falls (Falls—Number) analyses. Characteristics are provided for the full sample alongside subgroup characteristics stratified by recurrent fall status (<2 vs ≥2 falls) in the prior year. Continuous variables are presented as mean ± standard deviation (*SD*) or median [interquartile range (*IQR*)], as appropriate, with categorical variables presented as count (*n*) and proportion (%). Subgroup comparisons (*p*-values) were derived via independent-samples (*t*-tests) for normally distributed continuous variables, Mann–Whitney *U*-tests for nonnormally distributed continuous variables, or Pearson’s Chi-square (*Χ*^2^) tests for categorical variables. All core demographic (denoted by^C^) and candidate covariates are included, with ^N^denoting a candidate covariate retained in the Falls-Number analyses.

Mean ESS score was 7.0 ± 3.6, with 16.6% of the sample exceeding the threshold for EDS (ESS > 10). Median SIQ score was 30.0 [IQR: 25.0, 37.0], and 24.5% of the sample exceeded the upper-quartile threshold for ESI (SIQ > 37). There was a weak-to-moderate positive correlation between ESS and SIQ score (*r* = 0.30; *p* < .001). Approximately one-quarter of the sample exceeded the upper-quartile derived thresholds for elevated SIQ Cognitive (>9; 23.2%), Physiological (>15; 23.5%), and Responses to Sleep Inertia (>9; 23.2%) subscales, whereas a smaller proportion exceeded the elevated threshold for the Emotional subscale (>4; 15.3%).

Average habitual sleep duration was 7.5 ± 1.1 h, about one-third of the sample (33.2%) reported a history of sleep apnea, and the average modified three-item reduced morningness-eveningness questionnaire score (10.7 ± 2.8) suggested a “neutral” to “somewhat morning” circadian preference [38]. Slightly lower than one-fifth of the sample reported current sedative medication use (17.9%), and one-quarter of the sample (25.0%) reported a history of memory concerns.

Across subgroup comparisons, participants with recurrent falls had a significantly higher BMI (*p* = .03), prevalence of injurious falls (*p* < .001), and prevalence of memory concerns (*p* = .04), as well as longer habitual sleep duration (*p* = .008), relative to those without recurrent falls. Participants with recurrent falls also reported significantly greater daytime sleepiness, both by total ESS score (*p* = .01) and proportion of EDS (*p* = .03), as well as more severe sleep inertia, indicated by higher total SIQ score (*p* = .002) and proportion of ESI (*p* = .005). For SIQ subscales, Cognitive (*p* = .003) and Physiological (*p* = .001) scores were significantly higher among participants with recurrent falls, as were the proportions of participants with elevated Cognitive (*p* = .02) and Physiological (*p* = .001) sleep inertia symptoms. Additional medical, psychiatric, substance use, physical, and cognitive characteristics stratified by recurrent fall status are summarized in Table 2.

#### Falls-Injury sample characteristics

Characteristics for the Falls-Injury analytic sample are presented in Table 3, including characterization of the overall sample and a subgroup comparison based on stratification by injurious fall status (no vs yes). Overall, the Falls-Injury sample (*n* = 162) was generally similar to the larger Falls-Number sample; however, as expected, a greater proportion reported recurrent falls (54.9%) and at least one injurious fall (49.4%). Median SIQ (31.0 [IQR: 26.0, 39.0]) and mean ESS (7.5 ± 3.9) scores were modestly higher in this analytic subsample, as was the prevalence of ESI (29.6%) and EDS (21.6%). The correlation between ESS and SIQ was also modestly higher in this subsample, while still weak-to-moderate (*r* = 0.35, *p* < .001). The prevalence of sleep apnea history (37.0%) and sedative medication use (21.0%) was also modestly higher.

**Table 3:** Characteristics of the falls-injury analytic sample

Characteristic	Full sample	No injurious fall	≥1 Injurious fall	*P*
**Sample size**	162	82	80	-----
**Demographics**				
^C^Age (years), mean ± SD	75.4 ± 6.6	75.7 ± 6.7	75.1 ± 6.6	0.59
^C^Sex (female), *n* (%)	73 (45.1)	28 (34.1)	45 (56.2)	0.008
^C^Body mass index (kg/m^2^), mean ± SD	31.7 ± 7.7	30.5 ± 7.1	32.8 ± 8.1	0.06
^C^Education history (high school or less), *n* (%)	32 (19.8)	8 (9.8)	24 (30.0)	0.002
Race/ethnicity (white), *n* (%)	157 (96.9)	79 (96.3)	78 (97.5)	1.00
**Falls (prior year)**				
Recurrent falls (≥ 2), *n* (%)	89 (54.9)	42 (51.2)	47 (58.8)	0.42
Injurious fall (yes), *n* (%)	80 (49.4)	-----	-----	-----
**Daytime sleepiness and sleep inertia**				
Epworth Sleepiness Scale				
Total score, mean ± SD	7.5 ± 3.9	7.7 ± 3.8	7.3 ± 4.1	0.54
Excessive (>10), *n* (%)	35 (21.6)	16 (19.5)	19 (23.8)	0.64
Sleep Inertia Questionnaire (SIQ)				
Total score, median [IQR]	31.0 [26.0, 39.0]	29.5 [25.0, 36.0]	34.0 [28.0, 41.0]	0.004
Elevated (>37), *n* (%)	48 (29.6)	17 (20.7)	31 (38.8)	0.02
SIQ Subscale—Cognitive				
Total score, median [IQR]	7.0 [5.2, 10.0]	7.0 [5.0, 9.0]	8.0 [6.0, 10.0]	0.01
Elevated (>9), *n* (%)	42 (25.9)	12 (14.6)	30 (37.5)	0.002
SIQ Subscale—Physiological				
Total score, median [IQR]	12.0 [10.0, 16.0]	11.0 [9.0, 15.0]	14.0 [11.0, 17.2]	0.001
Elevated (>15), *n* (%)	52 (32.1)	19 (23.2)	33 (41.2)	0.02
SIQ Subscale—Emotional				
Total score, median [IQR]	3.0 [3.0, 4.0]	3.0 [3.0, 4.0]	3.0 [3.0, 4.0]	1.00
Elevated (>4), *n* (%)	24 (14.8)	9 (11.0)	15 (18.8)	0.24
SIQ Subscale—Response to sleep inertia				
Total score, median [IQR]	7.5 [6.0, 9.0]	7.0 [6.0, 9.0]	8.0 [6.0, 10.0]	0.10
Elevated (>9), *n* (%)	39 (24.1)	17 (20.7)	22 (27.5)	0.41
**Sleep**				
^I^Habitual sleep duration (h), mean ± SD	7.6 ± 1.3	7.4 ± 1.1	7.7 ± 1.5	0.15
^I^Sleep apnea history (yes), *n* (%)	60 (37.0)	20 (24.4)	40 (50.0)	0.001
^I^Circadian preference (modified 3-item rMEQ score), mean ± SD	10.6 ± 2.7	10.3 ± 2.8	10.8 ± 2.5	0.22
Daytime napping frequency (days per week), median [IQR]	2.0 [0.0, 4.0]	1.0 [0.0, 4.0]	2.0 [0.0, 4.0]	0.33
**Medical**				
^I^Sedative medication use (yes), *n* (%)	34 (21.0)	13 (15.9)	21 (26.2)	0.15
^I^Arthritis history (yes), *n* (%)	96 (59.3)	40 (48.8)	56 (70.0)	0.01
Hypertension history (yes), *n* (%)	89 (55.6)	45 (55.6)	44 (55.7)	1.00
**Psychiatric**				
Depressive symptomatology (modified Zung score), mean ± SD	33.9 ± 8.0	33.5 ± 7.1	34.3 ± 8.8	0.54
^I^Trait anxiety symptomatology, median [IQR]	31.0 [24.2, 37.0]	29.0 [24.0, 36.8]	31.5 [26.0, 38.0]	0.14
**Substance use**				
Alcohol Intake (drinks per week), median [IQR]	1.0 [0.0, 5.0]	2.0 [0.0, 6.0]	1.0 [0.0, 4.0]	0.44
Caffeine Intake (drinks per day), median [IQR]	2.0 [1.0, 3.0]	2.0 [1.5, 3.0]	2.0 [1.0, 3.0]	0.48
Smoking history (former/current), *n* (%)	73 (45.1)	34 (41.5)	39 (48.8)	0.44
**Physical**				
Dominant hand grip strength (lbs)				
Female, mean ± SD	46.0 ± 12.1	47.4 ± 11.0	45.2 ± 12.7	0.46
Male, mean ± SD	73.2 ± 19.7	71.5 ± 18.8	75.8 ± 21.0	0.32
Standing balance (at-risk), *n* (%)	55 (34.2)	28 (34.1)	27 (34.2)	1.00
Regular exercise (no), *n* (%)	87 (54.0)	43 (53.1)	44 (55.0)	0.93
**Cognitive**				
Telephone interview for cognitive status score, mean ± SD	34.6 ± 3.1	34.6 ± 3.1	34.6 ± 3.1	0.98
Memory concerns history (yes), *n* (%)	48 (29.6)	21 (25.6)	27 (33.8)	0.34

rMEQ, Reduced Morningness-Eveningness Questionnaire; Zung, Zung Self-Rating Depression Scale.

Characteristics for the sample used in the fall-related injury (Falls-Injury) analyses. Characteristics are provided for the full sample alongside subgroup characteristics stratified by injurious fall status (no injurious fall vs ≥1 injurious fall) in the prior year. Continuous variables are presented as mean ± standard deviation (*SD*) or median [interquartile range (IQR)], as appropriate, with categorical variables presented as count (*n*) and proportion (%). Subgroup comparisons (*p*-values) were derived via independent-samples (*t*-tests) for normally distributed continuous variables, Mann–Whitney U-tests for nonnormally distributed continuous variables, or Pearson’s Chi-square (*Χ*^2^) tests for categorical variables. All core demographic (denoted by^C^) and candidate covariates are included, with ^I^denoting a candidate covariate retained in the Falls-Injury analyses.

Across subgroup comparisons, the injurious fall group had a significantly higher proportion of female participants (*p* = .008), education level of high school or less (*p* = .002), sleep apnea history (*p* = .001), and arthritis history (*p* = .01), relative to the group of participants without an injurious fall. Participants with an injurious fall also reported more severe sleep inertia, indicated by significantly higher total SIQ score (*p* = .004) and proportion with ESI (*p* = .02). For SIQ subscales, Cognitive (*p* = .01) and Physiological (*p* = .001) scores were significantly higher among participants with an injurious fall, as were the proportions of participants with elevated Cognitive (*p* = .002) and Physiological (*p* = .02) sleep inertia symptoms. Additional medical, psychiatric, substance use, physical, and cognitive characteristics stratified by injurious fall status are summarized in Table 3.

### Associations of sleep inertia and daytime sleepiness with recurrent falls over the prior year (Falls-Number)

Table 4 presents the results from the logistic regression models examining sleep inertia and daytime sleepiness as predictors of recurrent falls over the prior year.

**Table 4:** Associations of sleep inertia and daytime sleepiness with recurrent falls over the prior year

Predictor	Unadjusted		Minimally adjusted		Fully adjusted	
	OR (95% CI)	*P*	OR (95% CI)	*P*	OR (95% CI)	*P*
**SIQ**						
Continuous	1.04 (1.01–1.06)	.003	1.04 (1.01–1.07)	.002	1.04 (1.00–1.07)	.03
Categorical	2.14 (1.27–3.57)	.004	2.28 (1.34–3.88)	.003	2.00 (1.07–3.73)	.03
**ESS**						
Continuous	1.09 (1.02–1.16)	.01	1.08 (1.01–1.15)	.02	1.06 (0.99–1.14)	.10
Categorical	2.02 (1.12–3.59)	.02	2.00 (1.09–3.61)	.03	1.74 (0.92–3.23)	.09

Results from logistic regression analyses examining associations of sleep inertia and daytime sleepiness with recurrent falls (<2 vs ≥2 falls) across the prior year. Sleep inertia was assessed using the Sleep Inertia Questionnaire (SIQ), and daytime sleepiness was measured using the Epworth Sleepiness Scale (ESS). Both SIQ and ESS were analyzed as continuous and categorical variables. ESI was defined as SIQ > 37 with the categorical SIQ variable reflecting a comparison between the upper quartile in the sample vs participants from the other three quartiles (Q4 vs Q1–Q3). Excessive daytime sleepiness (EDS) was defined based on an established threshold (ESS > 10) [30, 31], with the categorical ESS variable reflecting a comparison between those with and without EDS (EDS vs no EDS). Results are presented from unadjusted, minimally adjusted, and fully adjusted models. Minimally adjusted models included demographic covariates (age, sex, body mass index, and education history). Fully adjusted models additionally included habitual sleep duration, history of sleep apnea, circadian preference, depressive symptomatology, trait anxiety symptomatology, standing balance ability, and history of memory concerns. Odds ratios (OR), 95% confidence intervals (CI), and *p* values correspond to the focal predictor in each model.

When modeled continuously, higher SIQ scores were significantly associated with increased odds of recurrent falls in unadjusted analyses (OR = 1.04, 95% CI = 1.01–1.06, *p* = .003), with statistical significance also observed in minimally and fully adjusted analyses (OR = 1.04, 95% CI = 1.00–1.07, *p* = .03). This same pattern was observed in categorical analyses. ESI was significantly associated with recurrent falls in unadjusted (OR = 2.14, 95% CI = 1.27–3.57, *p* = .004), minimally adjusted, and fully adjusted analyses (OR = 2.00, 95% CI = 1.07–3.73, *p* = .03). In sensitivity analyses exploring quartile-specific associations (using Q1 as reference), significantly increased odds of recurrent falls were observed exclusively in Q4 (Table S3), suggesting a potential severity threshold effect that is specific to individuals with the highest sleep inertia severity.

For daytime sleepiness, higher ESS scores were significantly associated with increased odds of recurrent falls in unadjusted analyses (OR = 1.09, 95% CI = 1.02–1.16, *p* = .01). Statistical significance was also observed in the minimally adjusted analysis, but not in the fully adjusted analysis (OR = 1.06, 95% CI = 0.99–1.14, *p* = .10). This pattern was consistent in categorical analyses. EDS was significantly associated with recurrent falls in unadjusted (OR = 2.02, 95% CI = 1.12–3.59, *p* = .02) and minimally adjusted analyses, but not in fully adjusted analyses (OR = 1.74, 95% CI = 0.92–3.23, *p* = .09).

In post hoc analyses co-modeling categorical SIQ and ESS, the associations were slightly attenuated relative to being modeled separately, but the overall pattern of results was not meaningfully changed (Table S5).

### Associations of sleep inertia and daytime sleepiness with injurious falls over the prior year (Falls-Injury)

Table 5 presents the results from the logistic regression models examining sleep inertia and daytime sleepiness as predictors of injurious falls over the prior year (Falls-Injury).

**Table 5:** Associations of sleep inertia and daytime sleepiness with injurious falls over the prior year

Predictor	Unadjusted		Minimally adjusted		Fully adjusted	
	OR (95% CI)	*P*	OR (95% CI)	p	OR (95% CI)	*P*
**SIQ**						
Continuous	1.05 (1.02–1.09)	.003	1.04 (1.00–1.08)	0.045	1.03 (0.98–1.08)	.24
Categorical	2.42 (1.22–4.94)	.01	2.10 (1.01–4.45)	0.048	1.98 (0.82–4.91)	.13
**ESS**						
Continuous	0.98 (0.90–1.06)	.53	0.98 (0.89–1.07)	0.61	0.96 (0.86–1.06)	.42
Categorical	1.28 (0.61–2.75)	.51	1.28 (0.55–2.97)	0.56	1.15 (0.43–3.08)	.78

Results from logistic regression analyses examining associations of sleep inertia and daytime sleepiness with injurious falls (no vs yes) across the prior year. Sleep inertia was assessed using the Sleep Inertia Questionnaire (SIQ), and daytime sleepiness was measured using the Epworth Sleepiness Scale (ESS). Both SIQ and ESS were analyzed as continuous and categorical variables. ESI was defined by the threshold derived in the primary analytic sample (SIQ > 37), with the categorical SIQ variable reflecting a comparison between those with and without ESI (ESI vs no ESI). Excessive daytime sleepiness (EDS) was defined based on an established threshold (ESS > 10) [30, 31], with the categorical ESS variable reflecting a comparison between those with and without EDS (EDS vs no EDS). Results are presented from unadjusted, minimally adjusted, and fully adjusted models. Minimally adjusted models included demographic covariates (age, sex, body mass index, and education history). Fully adjusted models additionally included habitual sleep duration, history of sleep apnea, circadian preference, trait anxiety symptomatology, sedative medication use, and history of arthritis. Odds ratios (OR), 95% confidence intervals (CI), and *p* values correspond to the focal predictor in each model.

When modeled continuously, higher SIQ scores were significantly associated with increased odds of injurious falls in unadjusted analyses (OR = 1.05, 95% CI = 1.02–1.09, *p* = .003), with statistical significance also observed in the minimally adjusted analysis but not in the fully adjusted analysis (OR = 1.03, 95% CI = 0.98–1.08, *p* = .24). This same pattern was observed in categorical analyses. ESI was significantly associated with injurious falls in unadjusted (OR = 2.42, 95% CI = 1.22–4.94, *p* = .01) and minimally adjusted analyses, but not in the fully adjusted analysis (OR = 1.98, 95% CI = 0.82–4.91, *p* = .13).

Across continuous and categorical analyses, no statistically significant associations were observed between daytime sleepiness and injurious falls.

In post hoc analyses co-modeling categorical SIQ and ESS, the results were materially unchanged relative to when modeled separately (Table S6).

### Exploratory associations between sleep inertia subscales and falls outcomes

Table 6 presents the associations between SIQ subscales and recurrent falls (Falls-Number). In unadjusted analyses, higher scores on the Cognitive (OR = 1.10, 95% CI = 1.02–1.18, *p* = .01) and Physiological (OR = 1.08, 95% CI = 1.03–1.14, *p* = .001) subscales were significantly associated with recurrent falls; however, in fully adjusted analyses, statistical significance was only observed for the Physiological subscale (OR = 1.08, 95% CI = 1.01–1.15, *p* = .02). This pattern was consistent in categorical analyses. Elevated Cognitive (OR = 1.92, 95% CI = 1.13–3.23, *p* = .02) and Physiological (OR = 2.49, 95% CI = 1.48–4.17, *p* < .001) symptoms were significantly associated with recurrent falls in unadjusted analyses; however, in fully adjusted analyses, statistical significance was only observed for elevated Physiological symptoms (OR = 2.11, 95% CI = 1.16–3.84, *p* = .02). Across continuous and categorical analyses, no statistically significant associations were observed for the Emotional or Responses subscales.

**Table 6:** Associations between sleep inertia questionnaire subscales and recurrent falls over the prior year

SIQ subscales	Unadjusted		Minimally adjusted		Fully adjusted	
	OR (95% CI)	*P*	OR (95% CI)	*P*	OR (95% CI)	*P*
**Cognitive**						
Continuous	1.10 (1.02–1.18)	.01	1.10 (1.02–1.19)	.01	1.07 (0.98–1.18)	.12
Categorical	1.92 (1.13–3.23)	.02	2.05 (1.19–3.50)	.01	1.60 (0.85–2.96)	.14
**Physiological**						
Continuous	1.08 (1.03–1.14)	.001	1.09 (1.03–1.15)	.001	1.08 (1.01–1.15)	.02
Categorical	2.49 (1.48–4.17)	<.001	2.52 (1.46–4.31)	<.001	2.11 (1.16–3.84)	.02
**Emotional**						
Continuous	1.04 (0.85–1.25)	.70	1.07 (0.87–1.30)	.50	0.94 (0.71–1.22)	.65
Categorical	1.18 (0.61–2.20)	.61	1.27 (0.65–2.40)	.48	0.86 (0.38–1.85)	.70
**Responses**						
Continuous	1.07 (0.99–1.15)	.11	1.08 (0.99–1.17)	.08	1.06 (0.96–1.15)	.24
Categorical	1.23 (0.70–2.10)	.47	1.31 (0.73–2.29)	.35	1.20 (0.65–2.17)	.56

Results from logistic regression analyses exploring associations between Sleep Inertia Questionnaire (SIQ) subscales and recurrent falls (<2 vs ≥2 falls) across the prior year. SIQ subscales included Cognitive, Physiological, Emotional, and Responses to Sleep Inertia (“Responses”), with each subscale analyzed both continuously and categorically. For each subscale, ESI symptoms were defined by the upper quartile (SIQ_Cognitive_ > 9, SIQ_Physiological_ > 15, SIQ_Emotional_ > 4, and SIQ_Responses_ > 9), with the categorical subscale variables reflecting a comparison between the upper quartile in the sample versus participants from the other three quartiles (Q4 vs Q1–Q3). Results are presented from unadjusted, minimally adjusted, and fully adjusted models. Minimally adjusted models included demographic covariates (age, sex, body mass index, and education history). Fully adjusted models additionally included covariates capturing habitual sleep duration, history of sleep apnea, circadian preference, depressive symptomatology, trait anxiety symptomatology, standing balance ability, and history of memory concerns. Odds ratios (OR), 95% confidence intervals (CI), and *p* values correspond to the focal predictor in each model.

Table 7 presents the associations between SIQ subscales and injurious falls (Falls-Injury). In unadjusted analyses, higher scores on the Cognitive (*OR* = 1.17, *95% CI* = 1.05–1.32, *p* = .005) and Physiological (*OR* = 1.11, *95% CI* = 1.04–1.20, *p* = .002) subscales were significantly associated with injurious falls; however, in fully adjusted analyses, no statistically significant associations were observed. In categorical analyses, elevated Cognitive (OR = 3.50, 95% CI = 1.67–7.73, *p* < .001) and Physiological (OR = 2.33, 95% CI = 1.19–4.65, *p* = .01) symptoms were significantly associated with injurious falls in unadjusted analyses; however, in fully adjusted analyses, statistical significance was only observed for elevated Cognitive symptoms (OR = 3.89, 95% CI = 1.49–10.94, *p* = .005). Across continuous and categorical analyses, no statistically significant associations were observed for the Emotional or Responses subscales.

**Table 7:** Associations between Sleep Inertia Questionnaire Subscales and Injurious Falls over the Prior Year

SIQ subscales	Unadjusted		Minimally adjusted		Fully adjusted	
	OR (95% CI)	*P*	OR (95% CI)	*P*	OR (95% CI)	*P*
**Cognitive**						
Continuous	1.17 (1.05–1.32)	.005	1.16 (1.03–1.34)	.02	1.14 (0.98–1.34)	.10
Categorical	3.50 (1.67–7.73)	<.001	4.12 (1.85–9.73)	<.001	3.89 (1.49–10.94)	.005
**Physiological**						
Continuous	1.11 (1.04–1.20)	.002	1.10 (1.02–1.19)	.02	1.07 (0.97–1.17)	.18
Categorical	2.33 (1.19–4.65)	.01	1.98 (0.96–4.17)	.07	1.75 (0.74–4.22)	.20
**Emotional**						
Continuous	1.12 (0.85–1.50)	.43	1.00 (0.74–1.39)	.98	0.84 (0.56–1.26)	.38
Categorical	1.87 (0.78–4.73)	.16	1.43 (0.55–3.84)	.46	0.89 (0.28–2.89)	.84
**Responses**						
Continuous	1.08 (0.97–1.20)	.15	1.02 (0.91–1.14)	.76	1.01 (0.88–1.15)	.90
Categorical	1.45 (0.70–3.03)	.31	0.96 (0.42–2.16)	.91	0.77 (0.30–1.93)	.57

Results from logistic regression analyses exploring associations between Sleep Inertia Questionnaire (SIQ) subscales and injurious falls (no vs yes) across the prior year. SIQ subscales included Cognitive, Physiological, Emotional, and Responses to Sleep Inertia (“Responses”), with each subscale analyzed continuously and categorically. For each subscale, elevated sleep inertia (ESI) symptoms were defined by the threshold derived in the primary analytic sample (SIQ_Cognitive_ > 9, SIQ_Physiological_ > 15, SIQ_Emotional_ > 4, and SIQ_Responses_ > 9), with the categorical subscale variables reflecting a comparison between those with and without ESI symptoms on that subscale (ESI vs no ESI). Results are presented from unadjusted, minimally adjusted, and fully adjusted models. Minimally adjusted models included demographic covariates (age, sex, body mass index, and education history). Fully adjusted models additionally included habitual sleep duration, history of sleep apnea, circadian preference, trait anxiety symptomatology, sedative medication use, and history of arthritis. Odds ratios (OR), 95% confidence intervals (CI), and *p* values correspond to the focal predictor in each model.

### Post hoc analyses: Moderating influence of age and sex

Tables S7 and S8 present the post hoc moderation analyses for age and sex, respectively. No statistically significant interactions between predictors and moderators were observed across falls outcomes.

## Discussion

Sleep inertia and daytime sleepiness are safety hazards that may contribute to fall risk in older adults. This study examined associations between sleep inertia, daytime sleepiness, and falls outcomes among older adult participants of the WSC, focusing on both recurrent falls and the occurrence of an injurious fall over the previous year. Using logistic regression analyses, higher SIQ scores and ESI (SIQ > 37) were associated with increased odds of recurrent falls across both unadjusted and fully adjusted analyses. Similarly, higher ESS scores and EDS (ESS > 10) were associated with recurrent falls in unadjusted analyses; however, both associations attenuated to non-significant in the fully adjusted analyses. For injurious falls, higher SIQ scores and ESI were associated with increased odds of an injurious fall in unadjusted analyses, yet these associations were not statistically significant in the fully adjusted analyses. Neither ESS scores nor EDS were associated with injurious falls across any analyses. In SIQ subscale analyses, Physiological and Cognitive sleep inertia symptoms were most strongly associated with fall outcomes, and outcome-specific patterns were observed. Physiological symptoms were more strongly linked to recurrent falls, whereas Cognitive symptoms were more strongly linked to injurious falls. Together, these findings suggest that sleep inertia may represent an important, under-recognized risk factor for falls in older adults.

We approached this investigation with the hypothesis that more severe sleep inertia would associate with increased odds for both recurrent and injurious falls. The findings were largely consistent with this hypothesis, implicating sleep inertia severity as a potentially important contributor to fall risk in older adults. It is important to note that the relationships with injurious falls attenuated to statistically nonsignificant in the fully adjusted models; however, the odds ratios were similar across unadjusted, minimally adjusted, and fully adjusted analyses. As such, it is possible that the loss of statistical significance may be a consequence of reduced statistical power related to the smaller sample size and the additional covariates used in the fully adjusted analyses, rather than a meaningful change in the underlying association.

The associations between sleep inertia and falls may reflect both the direct contribution of sleep inertia to fall risk as well as its potential role as an indicator of broader health-related vulnerabilities. On the one hand, the cognitive, physical, and psychological impairments associated with sleep inertia may directly increase vulnerability to falls during the transition from sleep to wakefulness. As such, older adults with more severe or prolonged sleep inertia may be at heightened risk for falls in the early morning or following nocturnal awakenings. On the other hand, more severe sleep inertia may be a consequence of broader health-related vulnerabilities that increase fall risk more generally, extending beyond the post-awakening period. For example, heightened sleep inertia severity can present as a symptom of sleep or psychiatric disorders [23], which may independently increase vulnerability to falls outside of the sleep inertia window. It is also possible that sleep inertia severity is related to neurocognitive or neurodegenerative conditions in older adults that are associated with increased fall risk [54, 55]. However, the characteristics of sleep inertia in these conditions are not well understood, potentially representing a fruitful area for further investigation.

Analyses of SIQ subscales suggested potentially important nuance in the relationship between sleep inertia symptoms and falls, with Physiological symptoms showing stronger associations with recurrent falls and Cognitive symptoms showing stronger associations with injurious falls. The Physiological subscale items predominantly assess physical impairments of sleep inertia, including balance, motor coordination and slowing, fatigue, and physical heaviness, with the results potentially suggesting that sleep inertia-related physical impairments contribute more directly to fall occurrence and recurrence. The Cognitive subscale captures characteristics of processing speed, vigilance, concentration, attentional lapses, and thought organization. In this sense, sleep inertia-related cognitive impairments may be more strongly related to whether a fall results in injury, potentially due to factors like attentional lapses, impaired judgment, and slowed reaction time that increase the likelihood of higher-risk fall scenarios and limit the ability to respond adaptively during a fall event.

For daytime sleepiness, the results largely provided support for an association with recurrent falls, generally aligning with prior research linking daytime sleepiness to increased fall frequency [16–18]. In contrast, there was no statistical evidence supporting an association between daytime sleepiness and a higher likelihood of injurious falls. Given the established role of daytime sleepiness as a safety hazard, this null finding was unexpected and may not have a clear explanation. One possible explanation is that daytime sleepiness may lead to behavioral adaptations, such as reduced activity levels or less engagement in higher-risk environments, which could potentially limit exposure to situations that increase the likelihood of injurious falls. From this perspective, individuals experiencing daytime sleepiness may still be at a higher risk for an injurious fall, but behavioral adaptations could potentially act as a mitigating factor. Further research is needed to better understand the relationship between daytime sleepiness and injurious falls, as well as factors that may influence this relationship.

There are limitations of this investigation that warrant consideration. The sample was predominantly white, and future work is needed to extend these findings to more racially and ethnically diverse populations. In addition, the cross-sectional study design is observational in nature, precluding the ability to infer causality. The dataset also did not include information on the timing of falls, limiting our ability to directly link sleep inertia severity to early morning falls specifically. Prospective research should strive to co-measure the timing of falls alongside sleep inertia characteristics to better determine whether sleep inertia may directly contribute to falls. Complementary to this point, the measures used to capture sleep inertia, daytime sleepiness, and falls history inquired across differing temporal windows, and future research should aim to concurrently track these constructs during overlapping timeframes. In addition, sleep inertia and daytime sleepiness were assessed using self-report questionnaires collected at a single time point. Future studies incorporating objective measurements (e.g. psychomotor vigilance task [56, 57]) and longitudinal assessments may provide a more comprehensive characterization of sleep inertia and daytime sleepiness severity. Moreover, the threshold used to define ESI symptoms (e.g. SIQ > 37) was derived from the investigation sample and may not generalize to other cohorts and populations. Lastly, since the number of falls variable was collected using categorical response options, we were unable to model fall counts as a continuous outcome, instead utilizing a dichotomized approach for modeling. Continuous outcomes may offer advantages over dichotomized variables, including greater statistical power and reduced risk of spurious associations [58].

This study examined associations between sleep inertia, daytime sleepiness, and falls in older adults of the WSC, with a specific focus on recurrent and injurious falls. Our primary findings highlight sleep inertia severity as a potentially important and under-recognized contributor to falls in older adults, particularly recurrent falls. In addition, the results provide some further support for a relationship between daytime sleepiness and increased fall occurrence; however, unexpectedly, daytime sleepiness did not associate with injurious falls. Secondary analyses suggested physiological and cognitive sleep inertia symptoms as most relevant to fall outcomes, with physiological symptoms more strongly associated with recurrent falls, while cognitive symptoms were more strongly associated with injurious falls. Overall, the findings from this investigation steer future research toward clarifying the causal role of sleep inertia in falls. Advancing this line of inquiry may implicate sleep inertia as a potential therapeutic target to enhance fall prevention and management.

## Supplementary Material

Cook_et_al_Sleep_Advances_2026_Supplementary_Materials_Final_Clean_zpag106

## Data Availability

The data utilized in this investigation will be shared on reasonable request to the corresponding author, and in concordance with required IRB approval.
