# Supplementary material for: Sleep inertia, daytime sleepiness, and falls in older adults of the Wisconsin Sleep Cohort
Source: Sleep Adv. 2026 Sep 29;7(4):zpag106. doi: 10.1093/sleepadvances/zpag106 (PMC13690004; doi:10.1093/sleepadvances/zpag106)
Supplement: Supplementary file 1 [file cook_et_al_sleep_advances_2026_supplementary_materials_final_clean_zpag106.docx]

**Supplementary Materials**

**Manuscript Title:** Sleep Inertia, Daytime Sleepiness, and Falls in Older Adults of The Wisconsin Sleep Cohort

**Authors and Affiliations:**

Jesse D. Cook^1^, Jodi H. Barnet^2^, Jessica J. Love^1,3^, Laurel A. Ravelo^2^, Annaliise J. Hayrynen^4^, Amanda Rasmuson^2^, Erika W. Hagen^2^, Paul E. Peppard^2^, and David T. Plante^1,3^

^1^ University of Wisconsin-Madison School of Medicine and Public Health, Department of Psychiatry, Madison, WI, USA

^2^ University of Wisconsin-Madison School of Medicine and Public Health, Department of Population Health Sciences, Madison, WI, USA

^3^ University of Wisconsin-Madison, Department of Psychology, Madison, WI, USA

^4^ Meadville Medical Center, Neurology, Meadville, PA, USA

**Corresponding Author:**

Jesse David Cook, PhD

[Jdcook4@wisc.edu](mailto:Jdcook4@wisc.edu)

6001 Research Park Blvd.

Madison, WI, 53719

ORCID ID: 0000-0003-1905-0262

**Supplementary Table 1. Associations Between Covariates (Core and Candidate) and Falls Outcomes**

| **Covariate** | **Falls-Number** | | **Falls-Injury** | | |
| --- | --- | --- | --- | --- | --- |
|  | ***OR (95% CI)*** | ***P*** | ***OR (95% CI)*** | ***P*** |  |
|  |  |  |  |  |  |
| **Core Demographics** |  |  |  |  |  |
| Age^C^ | 1.00 (0.97-1.04) | 0.94 | 0.98 (0.94-1.03) | 0.50 |  |
| Sex^C^ | 0.78 (0.48-1.26) | 0.31 | 2.38 (1.28-4.49) | 0.006 |  |
| Body Mass Index^C^ | 1.03 (1.00-1.07) | 0.04 | 1.04 (1.00-1.09) | 0.049 |  |
| Education History^C^ | 0.96 (0.51-1.71) | 0.89 | 3.88 (1.69-9.80) | 0.001 |  |
|  |  |  |  |  |  |
| **Candidate Covariates** |  |  |  |  |  |
| Habitual Sleep Duration^N-I^ | **1.39 (1.13-1.71)** | **0.001** | **1.16 (0.92-1.47)** | **0.22** |  |
| Sleep Apnea History^N-I^ | **1.42 (0.87-2.30)** | **0.16** | **3.10 (1.62-6.07)** | **< 0.001** |  |
| Circadian Preference^N-I^ | **0.95 (0.87-1.03)** | **0.21** | **1.08 (0.96-1.21)** | **0.22** |  |
| Daytime Napping Frequency | 0.96 (0.86-1.06) | 0.42 | 1.04 (0.92-1.19) | 0.52 |  |
| Sedative Medication Use^I^ | 1.14 (0.61-2.06) | 0.67 | **1.85 (0.87-4.10)** | **0.11** |  |
| Arthritis History^I^ | 0.89 (0.56-1.43) | 0.63 | **2.21 (1.18-4.19)** | **0.01** |  |
| Hypertension History | 0.85 (0.53-1.36) | 0.49 | 1.03 (0.55-1.91) | 0.93 |  |
| Depressive Symptomatology^N^ | **1.03 (1.00-1.06)** | **0.04** | 1.02 (0.98-1.06) | 0.36 |  |
| Trait Anxiety Symptomatology^N-I^ | **1.02 (1.00-1.05)** | **0.07** | **1.03 (1.00-1.07)** | **0.06** |  |
| Weekly Alcohol Intake | 1.01 (0.97-1.06) | 0.57 | 0.98 (0.93-1.04) | 0.56 |  |
| Daily Caffeine Intake | 0.99 (0.86-1.14) | 0.93 | 1.00 (0.83-1.21) | 0.97 |  |
| Smoking History | 1.02 (0.64-1.63) | 0.93 | 1.31 (0.71-2.42) | 0.39 |  |
| Dominant Hand Grip Strength | 1.00 (0.98-1.01) | 0.55 | 1.01 (0.99-1.03) | 0.52 |  |
| Standing Balance^N^ | **1.36 (0.81-2.25)** | **0.24** | 1.04 (0.54-1.98) | 0.91 |  |
| Regular Exercise | 1.05 (0.66-1.69) | 0.84 | 1.10 (0.60-2.04) | 0.75 |  |
| Global Cognitive Status | 0.98 (0.91-1.06) | 0.60 | 1.00 (0.90-1.10) | 0.99 |  |
| Memory Concerns History^N^ | **1.94 (1.16-3.22)** | **0.01** | 1.44 (0.74-2.82) | 0.29 |  |

*Note.* ^C^ denotes an *a priori* selected core demographic variable included across all minimally and fully adjusted analyses; ^N^ denotes retention for only the Falls-Number fully adjusted analyses; ^I^ denotes retention for only the Falls-Injury fully adjusted analyses; ^N-I^ denotes retention for both the Falls-Number and Falls-Injury fully adjusted analyses

**Supplementary Table 1.** Univariate associations between covariates (core and candidate) and falls outcomes (Falls-Number and Falls-Injury). Bolded statistics reflect associations that were below the conservative significance threshold (*p* ≤ 0.25) used to identify candidate covariates for retention alongside a priori core demographics in the fully adjusted models.

**Supplementary Table 2. Characteristics Across SIQ Quartiles**

| **Characteristic** | | **Q1**  **(SIQ = 21 – 25)** | **Q2**  **(SIQ = 26 – 30)** | **Q3**  **(SIQ = 31 – 37)** | **Q4**  **(SIQ > 37)** | ***p*** |
| --- | --- | --- | --- | --- | --- | --- |
| **Sample Size** | | 110 | 99 | 87 | 96 | ----- |
|  | |  |  |  |  |  |
| **Demographics** | |  |  |  |  |  |
| Age (years), mean ± SD | | 74.8 ± 6.0 | 73.5 ± 6.2 | 75.6 ± 7.1 | 74.4 ± 6.9 | 0.17 |
| Sex (female), n (%) | | 40 (36.4) | 54 (54.5) | 30 (34.5) | 52 (54.2) | 0.003 |
| Body Mass Index (kg/m^2^), mean ± SD | | 29.5 ± 5.5 | 32.0 ± 7.7 | 31.3 ± 7.3 | 31.8 ± 7.8 | 0.047 |
| Education History (high school or less), n (%) | | 17 (15.5) | 16 (16.2) | 16 (18.4) | 27 (28.1) | 0.09 |
| Race/Ethnicity (white), n (%) | | 103 (93.6) | 96 (97.0) | 86 (98.9) | 92 (95.8) | 0.28 |
|  | |  |  |  |  |  |
| **Falls (Prior Year)** | |  |  |  |  |  |
| Recurrent Falls (≥ 2), n (%) | | 19 (17.3) | 18 (18.2) | 19 (21.8) | 32 (33.3) | 0.03 |
| Injurious Fall (yes), n (%) | | 11 (10.0) | 21 (21.2) | 17 (19.5) | 30 (31.2) | 0.002 |
|  | |  |  |  |  |  |
| **Daytime Sleepiness and Sleep Inertia** | |  |  |  |  |  |
| Epworth Sleepiness Scale | |  |  |  |  |  |
|  | Total Score, mean ± SD | 5.7 ± 3.4 | 6.3 ± 3.1 | 8.1 ± 3.6 | 8.3 ± 3.8 | < 0.001 |
|  | Excessive (> 10), n (%) | 11 (10.0) | 10 (10.1) | 20 (23.0) | 24 (25.0) | 0.003 |
| Sleep Inertia Questionnaire (SIQ) | |  |  |  |  |  |
|  | Total Score, median [IQR] | 23.0 [22.0, 24.0] | 28.0 [27.0, 29.0] | 34.0 [32.0, 35.0] | 43.0 [40.0, 48.0] | < 0.001 |
|  | Elevated (> 37), n (%) | 0 (0.0) | 0 (0.0) | 0 (0.0) | 96 (100) | < 0.001 |
| SIQ Subscale – Cognitive | |  |  |  |  |  |
|  | Total Score, median [IQR] | 5.0 [5.0, 6.0] | 6.0 [5.0, 8.0] | 8.0 [6.0, 9.0] | 11.0 [10.0, 12.0] | < 0.001 |
|  | Elevated (> 9), n (%) | 0 (0.0) | 2 (2.0) | 16 (18.4) | 73 (76.0) | < 0.001 |
| SIQ Subscale – Physiological | |  |  |  |  |  |
|  | Total Score, median [IQR] | 9.0 [8.0, 9.0] | 11.0 [10.0, 12.0] | 13.0 [11.0, 14.5] | 18.0 [16.0, 21.2] | < 0.001 |
|  | Elevated (> 15), n (%) | 0 (0.0) | 1 (1.0) | 14 (16.1) | 77 (80.2) | < 0.001 |
| SIQ Subscale – Emotional | |  |  |  |  |  |
|  | Total Score, median [IQR] | 3.0 [3.0, 3.0] | 3.0 [3.0, 3.0] | 4.0 [3.0, 4.0] | 4.0 [3.0, 5.0] | < 0.001 |
|  | Elevated (> 4), n (%) | 1 (0.9) | 3 (3.0) | 19 (21.8) | 37 (38.5) | < 0.001 |
| SIQ Subscale – Response to Sleep Inertia | |  |  |  |  |  |
|  | Total Score, median [IQR] | 6.0 [5.0, 6.8] | 7.0 [6.0, 8.0] | 9.0 [7.0, 11.0] | 10.0 [9.0, 12.0] | < 0.001 |
|  | Elevated (> 9), n (%) | 0 (0.0) | 7 (7.1) | 32 (36.8) | 52 (54.2) | < 0.001 |
|  | |  |  |  |  |  |
| **Sleep** | |  |  |  |  |  |
| Habitual Sleep Duration (hours), mean ± SD | | 7.5 ± 1.0 | 7.4 ± 1.2 | 7.5 ± 1.1 | 7.4 ± 1.2 | 0.78 |
| Sleep Apnea History (yes), n (%) | | 25 (22.7) | 28 (28.3) | 33 (37.9) | 44 (45.8) | 0.003 |
| Circadian Preference (modified 3-item rMEQ score), mean ± SD | | 11.3 ± 2.6 | 11.0 ± 3.0 | 10.6 ± 2.8 | 10.1 ± 2.7 | 0.02 |
| Daytime Napping Frequency (days per week), median [IQR] | | 1.0 [0.0, 3.0] | 2.0 [0.5, 4.0] | 3.0 [1.0, 5.0] | 1.0 [0.0, 4.0] | 0.008 |
|  | |  |  |  |  |  |
| **Medical** | |  |  |  |  |  |
| Sedative Medication Use (yes), n (%) | | 16 (14.5) | 18 (18.2) | 13 (14.9) | 23 (24.0) | 0.29 |
| Arthritis History (yes), n (%) | | 47 (42.7) | 53 (53.5) | 48 (55.2) | 66 (69.5) | 0.002 |
| Hypertension History (yes), n (%) | | 55 (50.0) | 48 (48.5) | 45 (52.3) | 60 (63.8) | 0.13 |
|  | |  |  |  |  |  |
| **Psychiatric** | |  |  |  |  |  |
| Depressive Symptomatology (modified Zung score), mean ± SD | | 28.9 ± 5.8 | 31.9 ± 5.9 | 33.1 ± 6.9 | 38.6 ± 7.9 | < 0.001 |
| Trait Anxiety Symptomatology, median [IQR] | | 24.5 [22.0, 29.8] | 29.0 [25.0, 33.0] | 30.0 [25.0, 37.0] | 37.0 [28.0, 44.0] | < 0.001 |
|  | |  |  |  |  |  |
| **Substance Use** | |  |  |  |  |  |
| Alcohol Intake (drinks per week), median [IQR] | | 2.0 [0.0, 5.4] | 2.0 [0.0, 6.0] | 2.0 [0.0, 5.8] | 1.0 [0.0, 3.0] | 0.36 |
| Caffeine Intake (drinks per day), median [IQR] | | 2.0 [1.0, 3.0] | 2.0 [1.0, 3.0] | 2.0 [1.0, 3.0] | 2.0 [1.0, 3.0] | 0.43 |
| Smoking History (former/current), n (%) | | 40 (36.4) | 42 (42.4) | 44 (50.6) | 50 (52.1) | 0.09 |
|  | |  |  |  |  |  |
| **Physical** | |  |  |  |  |  |
| Dominant Hand Grip Strength (lbs) | |  |  |  |  |  |
|  | Female, mean ± SD | 47.5 ± 13.0 | 48.2 ± 10.8 | 48.5 ± 11.5 | 46.4 ± 13.9 | 0.85 |
|  | Male, mean ± SD | 79.1 ± 16.9 | 81.0 ± 17.6 | 75.4 ± 19.6 | 73.7 ± 26.1 | 0.25 |
| Standing Balance (at-risk), n (%) | | 22 (20.0) | 24 (24.2) | 27 (31.0) | 35 (36.5) | 0.045 |
| Regular Exercise (no), n (%) | | 57 (51.8) | 49 (50.0) | 51 (58.6) | 61 (63.5) | 0.20 |
|  | |  |  |  |  |  |
| **Cognitive** | |  |  |  |  |  |
| Telephone Interview for Cognitive Status Score, mean ± SD | | 35.2 (2.4) | 35.4 (3.1) | 34.4 (3.1) | 34.7 (3.0) | 0.07 |
| Memory Concerns History (yes), n (%) | | 19 (17.3) | 20 (20.2) | 21 (24.1) | 38 (39.6) | 0.001 |
|  | |  |  |  |  |  |

*Note.* rMEQ = Reduced Morningness-Eveningness Questionnaire; Zung = Zung Self-Rating Depression Scale.

**Supplementary Table 2.** Characteristics from the primary analytic sample (n = 393; “Falls-Number”) across sleep inertia questionnaire (SIQ) quartiles: Q1 (SIQ = 21 – 25), Q2 (SIQ = 26 – 30), Q3 (SIQ = 31 – 37), and Q4 (> 37). Continuous variables are presented as mean ± standard deviation (*SD*) or median [interquartile range (*IQR*)], as appropriate, with categorical variables presented as count (*n*) and proportion (%). Global comparisons across SIQ quartiles were conducted via one-way Analysis of Variance (ANOVA) for normally distributed continuous variables, Kruskal-Wallis tests for non-normally distributed continuous variables, or Pearson’s chi-square (*Χ*^2^) tests for categorical variables.

**Supplementary Table 3. Falls-Number Associations by SIQ Quartiles**

| **SIQ Quartiles** | ***n*** | **Unadjusted** | **Minimally Adjusted** | **Fully Adjusted** |
| --- | --- | --- | --- | --- |
| Main Effect (*p* value) | ----- | 0.03 | 0.02 | 0.19 |
|  |  |  |  |  |
| Comparisons |  |  |  |  |
| Q1 (21 – 25; Reference) | 110 |  |  |  |
| Q2 (26 – 30) | 99 | 1.06 (0.52-2.17) | 1.05 (0.50-2.18) | 1.02 (0.48-2.15) |
| Q3 (31 – 37) | 87 | 1.34 (0.66-2.73) | 1.24 (0.60-2.55) | 1.13 (0.53-2.40) |
| Q4 (> 37) | 96 | 2.39 (1.26-4.66)^ | 2.48 (1.28-4.92)^ | 2.12 (0.96-4.74) |

**Supplementary Table 3.** Results from the sensitivity analyses that explored associations with recurrent falls (“Falls-Number”) based on sleep inertia questionnaire (SIQ) quartile (Q1 – Q4), using Q1 as reference. The sample size (*n*) for each quartile group is provided alongside the odds ratio and 95% confidence interval for each quartile comparison. Minimally adjusted models include core demographics (age, sex, body mass index, and education history) as covariates, with the fully adjusted models additionally including habitual sleep duration, history of sleep apnea, circadian preference, depressive symptomatology, trait anxiety symptomatology, standing balance ability, and history of memory concerns. Statistical significance is denoted by * *p* < 0.05, ^ *p* < 0.01, ^#^ *p* < 0.001.

**Supplementary Table 4. Falls-Injury Associations by SIQ Severity Categories**

| **SIQ Severity Category  (Falls-Number Quartile Cutpoints)** | ***n*** | **Unadjusted** | **Minimally Adjusted** | **Fully Adjusted** |
| --- | --- | --- | --- | --- |
| Main Effect (*p* value) | ----- | 0.005 | 0.02 | 0.054 |
|  |  |  |  |  |
| Comparisons |  |  |  |  |
| Q1 (21 – 25; Reference) | 40 |  |  |  |
| Q2 (26 – 30) | 39 | 3.08 (1.23-8.07)* | 3.41 (1.26-9.75)* | 3.46 (1.19-10.60)* |
| Q3 (31 – 37) | 35 | 2.49 (0.97-6.66) | 2.48 (0.89-7.17) | 2.41 (0.75-8.05) |
| Q4 (> 37) | 48 | 4.81 (1.98-12.37)^#^ | 4.40 (1.69-12.18)^ | 4.53 (1.41-15.47)* |

**Supplementary Table 4.** Results from the sensitivity analyses that explored associations with injurious falls (“Falls-Injury”) based on sleep inertia questionnaire (SIQ) severity categories, using the lowest severity category (Q1) as reference. Thresholds for severity categories (Q1 – Q4) were carried forward from the Falls-Number sample and the distribution closely resembles a quartile split. The sample size (*n*) for each severity group is provided alongside the odds ratio and 95% confidence interval for each group comparison. Minimally adjusted models include core demographics (age, sex, body mass index, and education history) as covariates, with the fully adjusted models additionally including habitual sleep duration, history of sleep apnea, circadian preference, trait anxiety symptomatology, sedative medication use, and history of arthritis. Statistical significance is denoted by * *p* < 0.05, ^ *p* < 0.01, ^#^ *p* < 0.001.

**Supplementary Table 5. Independent Associations of Sleep Inertia and Daytime Sleepiness with Recurrent Falls: Mutually Adjusted Models**

| **Predictor** | | **SIQ + ESS** | | **Minimally Adjusted** | | **Fully Adjusted** | |
| --- | --- | --- | --- | --- | --- | --- | --- |
|  |  | ***OR* (95% *CI*)** | ***P*** | **OR (95% *CI*)** | ***P*** | ***OR* (95% *C*I)** | ***P*** |
| **SIQ – Categorical** | |  |  |  |  |  |  |
|  | ESI (> 37) | 2.01 (1.19-3.37) | 0.01 | 2.13 (1.24-3.65) | 0.007 | 1.99 (1.04-3.68) | 0.04 |
| **ESS – Categorical** | |  |  |  |  |  |  |
|  | EDS (> 10) | 1.84 (1.00-3.29) | 0.048 | 1.79 (0.96-3.26) | 0.07 | 1.70 (0.89-3.17) | 0.11 |

**Supplementary Table 5.** Results from post hoc logistic regression analyses examining associations of sleep inertia and daytime sleepiness with recurrent falls (< 2 vs. ≥ 2 falls) across the prior year, while co-modeling the predictors as categorical variables. Elevated sleep inertia was defined as SIQ > 37 with the categorical SIQ variable reflecting a comparison between the upper quartile in the sample versus participants from the other three quartiles (Q4 vs. Q1 – Q3). Excessive daytime sleepiness (EDS) was defined based on an established threshold (ESS > 10),^30,31^ with the categorical ESS variable reflecting a comparison between those with and without EDS (EDS vs. no EDS). Results are presented from a model that includes both the SIQ and ESS categorical predictors, as well as minimally adjusted and fully adjusted models. The minimally adjusted models included demographic covariates (age, sex, body mass index, and education history) with the two predictors, while the fully adjusted models additionally included habitual sleep duration, history of sleep apnea, circadian preference, depressive symptomatology, trait anxiety symptomatology, standing balance ability, and history of memory concerns. Odds ratios (*OR*), 95% confidence intervals (*CI*), and *p* values are presented for the focal predictors from each model.

**Supplementary Table 6. Independent Associations of Sleep Inertia and Daytime Sleepiness with Injurious Falls: Mutually Adjusted Models**

| **Predictor** | | **SIQ + ESS** | | **Minimally Adjusted** | | **Fully Adjusted** | |
| --- | --- | --- | --- | --- | --- | --- | --- |
|  |  | ***OR* (95% *CI*)** | ***P*** | **OR (95% *CI*)** | ***P*** | ***OR* (95% *C*I)** | ***P*** |
| **SIQ – Categorical** | |  |  |  |  |  |  |
|  | ESI (> 37) | 2.38 (1.19-4.89) | 0.01 | 2.06 (0.99-4.41) | 0.06 | 1.98 (0.82-4.90) | 0.13 |
| **ESS – Categorical** | |  |  |  |  |  |  |
|  | EDS (> 10) | 1.15 (0.53-2.51) | 0.71 | 1.16 (0.49-2.73) | 0.73 | 1.14 (0.42-3.10) | 0.79 |

**Supplementary Table 6.** Results from post hoc logistic regression analyses examining associations of sleep inertia and daytime sleepiness with injurious falls (no vs. yes) across the prior year, while co-modeling the predictors as categorical variables. Elevated sleep inertia was defined as SIQ > 37 with the categorical SIQ variable reflecting a comparison between the upper quartile in the sample versus participants from the other three quartiles (Q4 vs. Q1 – Q3). Excessive daytime sleepiness (EDS) was defined based on an established threshold (ESS > 10),^30,31^ with the categorical ESS variable reflecting a comparison between those with and without EDS (EDS vs. no EDS). Results are presented from a model that includes both the SIQ and ESS categorical predictors, as well as minimally adjusted and fully adjusted models. The minimally adjusted models included demographic covariates (age, sex, body mass index, and education history) with the two predictors, while the fully adjusted models additionally included habitual sleep duration, history of sleep apnea, circadian preference, trait anxiety symptomatology, sedative medication use, and history of arthritis. Odds ratios (*OR*), 95% confidence intervals (*CI*), and *p* values are presented for the focal predictors from each model.

**Supplementary Table 7. Moderating Role of Age**

| **Predictor** | | **Falls-Number** | | | **Falls-Injury** | | |
| --- | --- | --- | --- | --- | --- | --- | --- |
|  |  | ***OR*** | ***95% CI*** | ***P*** | ***OR*** | ***95% CI*** | ***P*** |
| ESS | | 0.99 | (0.98 – 1.00) | 0.12 | 1.01 | (0.99 – 1.02) | 0.47 |
| SIQ | | 1.00 | (1.00 – 1.00) | 0.79 | 1.00 | (0.99 – 1.01) | 0.86 |
| SIQ Subscales | |  |  |  |  |  |  |
|  | Cognitive | 1.00 | (0.99 – 1.01) | 0.80 | 1.00 | (0.98 – 1.02) | 0.80 |
|  | Physiological | 1.00 | (0.99 – 1.01) | 0.95 | 1.00 | (0.98 – 1.01) | 0.73 |
|  | Emotional | 0.98 | (0.94 – 1.01) | 0.16 | 0.98 | (0.93 – 1.03) | 0.49 |
|  | Responses | 0.99 | (0.98 – 1.00) | 0.22 | 1.00 | (0.98 – 1.02) | 0.93 |

**Supplementary Table 7.** Results from the post hoc analyses assessing the moderating effect of age on associations between predictors and falls outcomes. Predictors include Epworth Sleepiness Scale (ESS), Sleep Inertia Questionnaire (SIQ), and SIQ Subscales (Cognitive, Physiological, Emotional, and Responses to Sleep Inertia [Responses]). All predictors and age were modeled continuously. Odds ratios (*OR*), 95% confidence intervals (*CI*), and *p* values are reported for the interaction term from the fully adjusted models.

**Supplementary Table 8. Moderating Role of Sex**

| **Predictor** | | **Falls-Number** | | | **Falls-Injury** | | |
| --- | --- | --- | --- | --- | --- | --- | --- |
|  |  | ***OR*** | ***95% CI*** | ***P*** | ***OR*** | ***95% CI*** | ***P*** |
| ESS | | 0.98 | (0.85 – 1.12) | 0.73 | 0.96 | (0.79 – 1.17) | 0.70 |
| SIQ | | 1.02 | (0.97 – 1.08) | 0.37 | 0.99 | (0.91 – 1.08) | 0.86 |
| SIQ Subscales | |  |  |  |  |  |  |
|  | Cognitive | 1.10 | (0.94 – 1.30) | 0.22 | 1.19 | (0.88 – 1.65) | 0.26 |
|  | Physiological | 1.01 | (0.91 – 1.12) | 0.89 | 0.98 | (0.83 – 1.16) | 0.82 |
|  | Emotional | 1.16 | (0.74 – 1.84) | 0.52 | 0.96 | (0.48 – 1.92) | 0.90 |
|  | Responses | 1.16 | (0.98 – 1.38) | 0.09 | 0.93 | (0.71 – 1.22) | 0.61 |

**Supplementary Table 8.** Results from the post hoc analyses assessing the moderating effect of sex on associations between predictors and falls outcomes. Predictors include Epworth Sleepiness Scale (ESS), Sleep Inertia Questionnaire (SIQ), and SIQ Subscales (Cognitive, Physiological, Emotional, and Responses to Sleep Inertia [Responses]). All predictors were modeled continuously, with sex modeled categorically (male vs. female). Odds ratios (*OR*), 95% confidence intervals (*CI*), and *p* values are reported for the interaction term from the fully adjusted models.

**Supplementary Figure 1. Age- and Sex-Stratified Sleep Inertia Questionnaire Subscale Scores**

**Supplementary Figure 1.** Boxplots displaying total scores for the four Sleep Inertia Questionnaire subscales: Cognitive (Panel A), Physiological (Panel B), Emotional (Panel C), and Responses to Sleep Inertia (Responses; Panel D). Plots are stratified by age group (60–69, 70–79, and 80+) and participant sex (Male [blue], Female [orange]). The horizontal line within each box indicates the median, while box bounds represent the interquartile range (IQR; 25th and 75th percentiles) and the whiskers extend no further than 1.5 × IQR. Individual jittered points represent participant scores.
